# Towards agile large-scale predictive modelling in drug discovery with flow-based programming design principles

**DOI:** 10.1186/s13321-016-0179-6

**Published:** 2016-11-24

**Authors:** Samuel Lampa, Jonathan Alvarsson, Ola Spjuth

**Affiliations:** 1 Department of Pharmaceutical Biosciences, Uppsala University, Box 591, 751 24 Uppsala, Sweden; 2 Science for Life Laboratory, Uppsala University, Box 3037, 750 03 Uppsala, Sweden

**Keywords:** Predictive modelling, Machine learning, Workflows, Drug discovery, Flow-based programming

## Abstract

Predictive modelling in drug discovery is challenging to automate as it often contains multiple analysis steps and might involve cross-validation and parameter tuning that create complex dependencies between tasks. With large-scale data or when using computationally demanding modelling methods, e-infrastructures such as high-performance or cloud computing are required, adding to the existing challenges of fault-tolerant automation. Workflow management systems can aid in many of these challenges, but the currently available systems are lacking in the functionality needed to enable agile and flexible predictive modelling. We here present an approach inspired by elements of the flow-based programming paradigm, implemented as an extension of the Luigi system which we name SciLuigi. We also discuss the experiences from using the approach when modelling a large set of biochemical interactions using a shared computer cluster.

**Graphical abstract:**
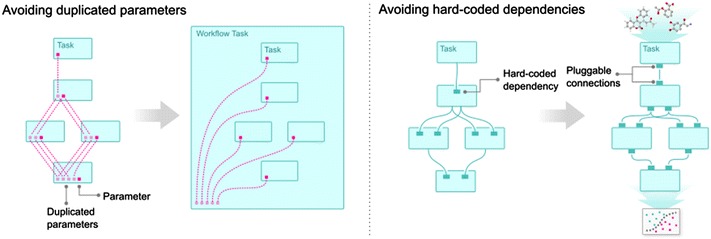
.

.

## Background

Predictive modelling is widely used in drug discovery with applications including prediction of interaction, inhibition and toxicity [1–3]. In ligand-based approaches, such as quantitative structure-activity relationship (QSAR), machine learning is commonly used to correlate chemical structures with activity while ligands are described numerically using descriptors [4]. Such modelling efforts consist of a set of computational tasks that are commonly invoked manually or with shell scripts that glue together multiple tasks into a simple form of pipeline. A computational task not uncommon in machine learning for drug discovery is a set of cross-validations nested with parameter sweeps to find optimal parameters for the model training. Such intricate sets of computations create complex task dependencies that are not always easy to encode in existing tools, if at all possible. Furthermore, as data sizes increase there is a need to use high-performance e-infrastructures such as compute clusters or cloud resources to carry out analyses. These add their own requirements, making reproducible, fault-tolerant automation even more difficult to achieve [5].

Scientific workflow management systems (WMS) are a possible solution in this context as they provide improved maintainability and robustness to failure over plain shell scripts. They provide this by describing the set of computations, the data they use and the dependencies between them in a generic way. Lower level details such as the logistics of data handling and task scheduling are left to the WMS. By hiding such technical details, they allow the researcher to focus on the research problem at hand when authoring the workflow rather than getting bogged down with peripheral matters. Thus, modifying the workflow connectivity becomes less complex and error-prone.

Commonly used workflow tools for predictive modelling in drug discovery include KNIME [6, 7] and PipelinePilot [8], where KNIME is an open source software with proprietary extensions and PipelinePilot is a proprietary software application. Both provide user interaction via a graphical user interface (GUI) where researchers can drag and drop components and build workflows for predictive modelling, among other things. While a GUI has clear advantages over text-based user interfaces for scientists lacking expertise in scripting or programming, it is unclear whether it provides any advantages over text-based user interfaces in terms of efficiency for expert users [9]. Graphical rich clients typically put more requirements on the computer on which they are run, such as requiring a graphical desktop system, which is not always available on HPC systems. This means that the tool can not be deployed fully to such HPC systems. Instead, the graphical client has to be run on the user’s local computer even when the jobs are executed remotely. Also, even when a graphical desktop system is available on an HPC system, performance reasons might make it impractical to access a graphical client over a secure shell (SSH) connection, as is often needed.

KNIME, by being the only open source tool of the mentioned tools, might be considered a good default choice for the types of use cases discussed in this study. However, the open source version of KNIME does not support HPC (“remote execution”), creation of libraries of custom, re-usable components (“custom node repository”) or detailed audit logging [10], all of which are features of vital importance to the use cases discussed in this paper. See Table 1 for a comparison between KNIME and the solution presented in this paper.

**Table 1:** Feature comparison: KNIME open source versus “Vanilla” Luigi and SciLuigi

Feature	KNIME open source	Luigi	SciLuigi
Authoring interface	GUI (rich client)	Text / CLI	Text / CLI
Implementation language	Java	Python	Python
Scheduling mode	Independent threads	Pull	Pull
HPC support	No	No	Yes
Custom re-usable components	No	No	Yes
Audit trail	No	No	Yes
Sub-workflows	Yes	No	No
Named ports	Yes	No	Yes
Nested loops	Yes	No	Yes
Interactive workflow debugging	Partly	Yes	Yes
CLI tool integration	Yes	Yes	Yes
Stream processing	Yes	No	No
Graphical workflow visualization	Yes	Yes	Yes
Supports scripting	Yes	Yes	Yes

In the wider field of bioinformatics there are numerous scientific workflow tools available for analysis, e.g., in genomics and proteomics [11], but a big proportion of these tools have various characteristics that limit their usefulness in highly complex analyses, such as when combining cross-validation and parameter sweeps. Furthermore, some tools do not support defining custom, re-usable components that can be assembled *ad hoc* for new workflows. In many WMS tools, complex workflows cannot be created without combining the workflow tool with shell scripts [12], pointing to their limitations for complex use cases.

Galaxy [13–15] and Yabi [16] are GUI-centric tools or frameworks with a client/server architecture that require the installation of a server daemon and meta data to support automatic GUI generation. By their GUI-centric nature, they do not allow a level of programmability similar to the text-based tools, meaning that it is not equally easy to use programmatic constructs such as loops to automate repetitive workflow patterns such as parameter sweeps. Galaxy supports a REST-interface [17] that can be used to provide this type of programmability, but this requires interfacing the tool with external scripts outside of the tool itself.

Snakemake [18], NextFlow [19] and BPipe [20] are text-based tools implemented as Domain Specific Languages (DSL). DSLs are mini-languages created specifically for the need of a specific domain [21], such as the topic at hand, scientific workflows. While DSLs can simplify workflow writing by allowing the workflows to be defined in a language that more closely maps to the problem at hand [22], they often impose limits on the types of workflows that can easily be modelled without having to modify the language itself [23]. They also often require *ad hoc* solutions for integrating with existing version control software, editors and debuggers [21]. Thus, DSLs can be too limiting for highly complex workflow constructs such as those in machine learning for drug discovery. This was perceived to be the case with Snakemake and BPipe. NextFlow’s DSL allows more flexibility due to its dataflow-based implementation, but does not support creating a library of reusable component definitions. Instead, NextFlow requires components to be defined in conjunction with the workflow definition [24].

Ruffus [25] and Luigi [26] are text-based tools exposed as programming libraries, meaning that their functionality is supposed to be used from within an existing scripting language such as Python. As programming libraries, they generally require more code for defining workflows compared to DSL tools but on the other hand provide greater flexibility, as they allow users to make use of the full power of the generic programming language in which they were implemented [27].

**Fig. 1:**
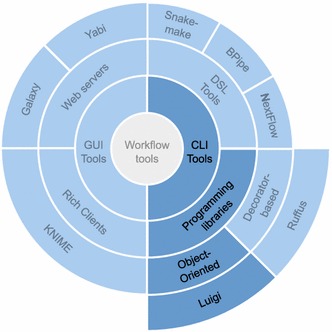
Sunburst diagram showing the hierarchical structure of the workflow tool landscape and Luigi’s position therein

While Ruffus provides an API based on decorators, Luigi provides an object-oriented programming API, which can be perceived as more familiar to some developers. Luigi also allows more control over output file naming than Ruffus. Furthermore it has support for the Apache Hadoop [28] and Apache Spark [29] execution environments together with support for the local file system in the same framework. Figure 1 gives an overview over Luigi’s relation to other workflow tools. Luigi thus was perceived as one of the most promising tools for use in the type of analyses described in this paper.

Despite these advantages, Luigi has shortcomings in some areas that can lead to brittle and hard-to-maintain workflow code limiting its applicability to complex analyses in drug discovery.

Flow-based programming (see the “Methods” section for details) is a paradigm developed for general purpose programs, suggesting a set of core design principles for achieving robust yet easy to modify component-oriented systems—a good description of what scientific workflow systems are aimed to be.

With this in mind, we present below a solution for agile development of highly complex workflows in machine learning for drug discovery, based on selected design principles from flow-based programming combined with the Luigi workflow framework, which we have named SciLuigi. In addition, functionality commonly used in scientific workflows has been added that was not included in vanilla Luigi, such as support for an HPC resource manager and audit logging capabilities.

The solution is demonstrated on a machine learning problem for modelling a large set of biochemical interactions using a shared computer cluster. Note that evaluating the actual modelling, and evaluation of the performance thereof, is outside the scope of this article, which is instead focused on solutions for the automation and coordination of such workflows, rather than the computational modelling methods themselves.

## Results

Agile development of complex workflows in machine learning for drug discovery requires adequate workflow management tools that support the complexity of these analyses. To this end, we have developed a solution based on the Luigi workflow library.

Compared with KNIME, the SciLuigi solution presented here provides three additions of vital importance for machine learning workflows in drug discovery: HPC support, ability to create a library of custom, re-usable components and detailed audit trails. See Table 1 for a detailed point-by-point comparison between KNIME, Luigi and SciLuigi.

As described in detail in the “Methods” section, Luigi has a number of limitations in relation to complex analyses for machine learning in drug discovery. To overcome these limitations, we extended Luigi with a selected set of design principles from the flow-based programming paradigm in an improved API. The resulting solution was packaged into a programming library named SciLuigi, which is available as open source on GitHub [30].

A summary of how the limitations in Luigi were solved in the SciLuigi approach is shown below.

### Separation of workflow definition from tasks

**Fig. 2:**
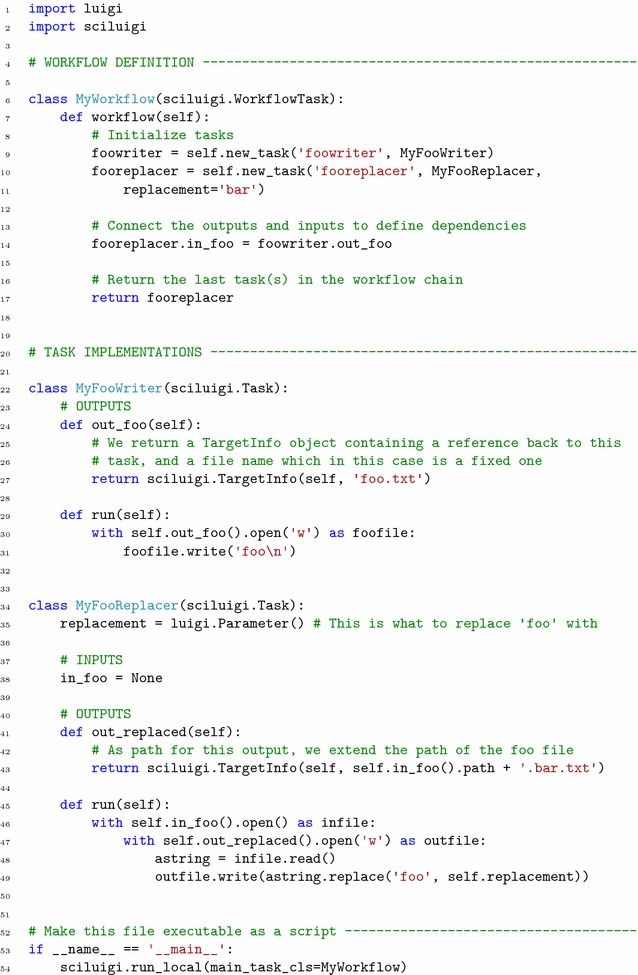
Code example of a simple workflow and tasks defined in SciLuigi. Out-port fields are functions that return a TargetInfo object, containing all info needed to retrieve both the target (file) with the data, as well as the task that produced it. In-port fields are assigned TargetInfo-returning methods from upstream tasks in the workflow definition, which is why we can write code that uses them in the out-port and run methods. See *line 18* for how the workflow connectivity is defined, by assigning the output of an out-port to an in-port

Agile workflow design requires the ability to quickly re-wire workflow connectivity. This is not easily done if dependencies are hard-coded inside task definitions, as is done in Luigi. Thus, inspired by the flow-based programming principle of *separate network definition* (see “Methods” section for details) we developed a programming API in SciLuigi that enables tasks to be instantiated and connected without changing their internal definition. See Fig. 2 for a code example demonstrating this.

### Avoiding parameter duplication

Another problem hindering agile workflow design in Luigi is unnecessary parameter duplication. Since upstream tasks in Luigi are instantiated inside the downstream tasks that depend on them, parameters have to be defined in all tasks downstream of the task in which they are used, just to forward their values. This creates an exponentially increasing amount of API dependencies between tasks that are not closely related. For large and complex workflows, this substantial maintenance overhead hinders agile workflow development. We have solved this in SciLuigi by embedding the workflow definition code in a workflow object which subclasses Luigi’s Task class.

**Fig. 3:**
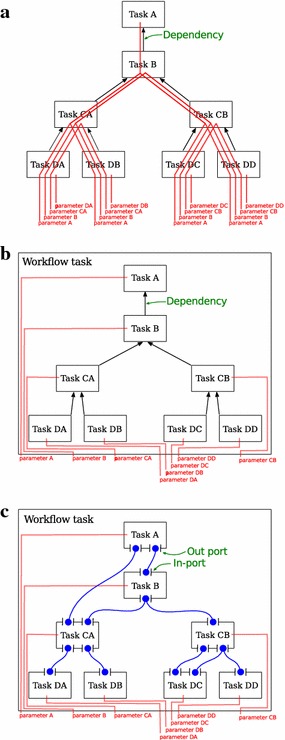
Solving the problem of duplicated parameters. This figure shows: **a** How parameters are defined and forwarded in vanilla Luigi; **b** How parameters are defined and forwarded in SciLuigi, using a wrapping workflow task; and **c** How SciLuigi also defines dependencies between the data on in- and out-ports rather than only directly between tasks. Commenting in more detail we see in **a** how every parameter definition has to be repeated for every downstream task, from where it was first defined. In **b**, we see that when the workflow is wrapped in a task, parameters only need to be duplicated once (in the workflow task). In **c**, we see how—in SciLuigi—dependencies are defined between in-ports and out-ports instead of directly between tasks. This means that task-specific information about names or positions of out-ports of upstream tasks are not contaminating downstream tasks

This allows inputs to be defined on the workflow object and for parameter values to be passed directly to the tasks that use them. Unnecessary dependencies between tasks, and duplicated code, are thus avoided, resulting in a more agile workflow development. See Fig. 3 for an illustration of this problem and how it is solved in SciLuigi.

### Workflow definition in terms of data—not task dependencies

**Fig. 4:**
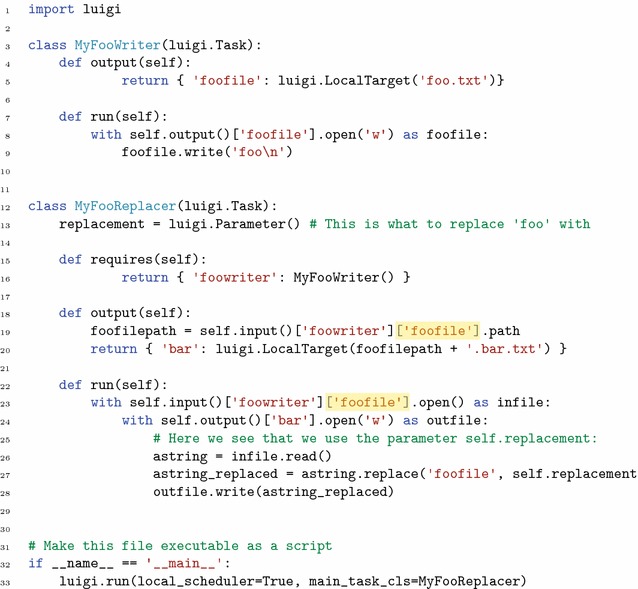
Code example showing two tasks connected into a simple workflow in vanilla Luigi. The task MyFooReplacer depends on MyFooWriter. Note that there is no central workflow definition, but that dependencies are specified within individual tasks in their requires() method (in this case only in the MyFooReplacer). The parts highlighted with yellow in MyFooReplacer on lines 19 and 23 contain information that is specific to the upstream task MyFooWriter. This means that MyFooReplacer is not independent from this upstream task, and can thus not be connected to other upstream tasks without modifying its internal code

For scientific workflows it is important to be able to specify task dependencies in terms of data and not only in terms of the finished execution of upstream tasks. This is because bioinformatics tools commonly produce or consume more than one data set at a time, making it important to capture this level of detail in the dependency specification. If not captured in the workflow definition, this logic needs to be captured in custom logic inside task definitions, creating hidden dependencies between tasks and hindering agile workflow development. See Fig. 4 for an example of this.

To meet this need, we have developed an approach inspired by the principle of *named ports* from flow-based programming, in which each input and output of a workflow component is given a name and the workflow dependencies are defined between such pairs of inputs and outputs rather than between tasks. See Fig. 3c for an illustration of this.

In detailed terms, task outputs in SciLuigi are defined by implementing methods with a special naming scheme starting with out_ and followed by a unique name. These methods return an object (of type TargetInfo), which keeps track of both the name of the file created for that output and a reference to the task that produced the output. Inputs are similarly defined by fields with a naming scheme starting with in_ followed by a unique name. Inputs are plain fields while outputs are implemented as methods. When developing workflows, these output methods are connected to the input fields of downstream tasks with a syntax similar to variable assignment. Downstream tasks will use the file name in the received (TargetInfo) object to find the data it needs as input and the reference to the upstream task to provide information about tasks it depends on. See Fig. 2 for a code example demonstrating this.

### Automatic audit information

In scientific workflows it is important to have a complete track record of what has been executed, including the command name, parameter values and execution times. Since Luigi lacks this feature out-of-the-box, we have extended Luigi with auditing functionality that stores important data about each execution of a task in a structured and easy to parse data format. This information is kept separate from the normal log function in Luigi to enable improved machine-readability.

### Helper functionality for running shell commands as batch HPC jobs

Scientific workflows are commonly executed on e-infrastructures, such as High-Performance Computing (HPC) clusters, as well as on users’ local computers. To support this we have extended Luigi with helper methods that allow the choice between executing shell commands either as HPC jobs or on the local computer, based on a configuration parameter passed to the task.

### Case study

In order to demonstrate the features of SciLuigi, we applied it to an example QSAR modelling application.

#### Introduction

The LIBLINEAR software [31] constitutes a fast SVM implementation based on linear SVM. This case study is set up as a small study of the effect of training set size on modelling time and model performance. These kinds of studies can result in non-trivial workflows due to nested cross-validation and parameter sweeps needed to properly tune model parameters and evaluate model performance.

#### Materials and methods

**Fig. 5:**
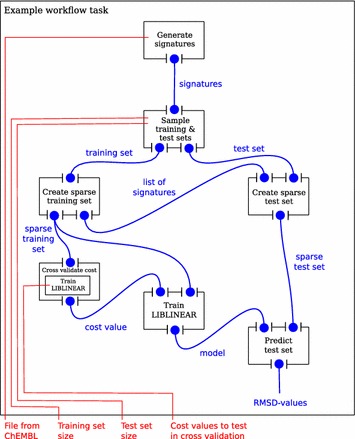
A representative part of the real life workflow from the example application. Note in particular the output “sparse training set” that is used in multiple downstream locations. In other words, the same data set is used by multiple processes in the workflow

We trained QSAR models using the LIBLINEAR software [31] with molecules described by the signature descriptor [32]. For linear SVM, the *cost* parameter needs to be tuned. We tested 15 values (0.0001, 0.0005, 0.001, 0.005, 0.01, 0.05, 0.1, 0.25, 0.5, 0.75, 1, 2, 3, 4, 5) in a 10-fold, cross-validated parameter sweep. Five different training set sizes (500, 1000, 2000, 4000, 8000) were tested and evaluated with a test set size of 1000. The data set consisted of 10,000 logarithmic solubility values chosen randomly from the 37,099 data points that were not given as ’larger than’ in a data set from Pubchem [33]. SciLuigi was used to design and orchestrate the workflow using the components schematically outlined in Fig. 5.

#### Results

**Fig. 6:**
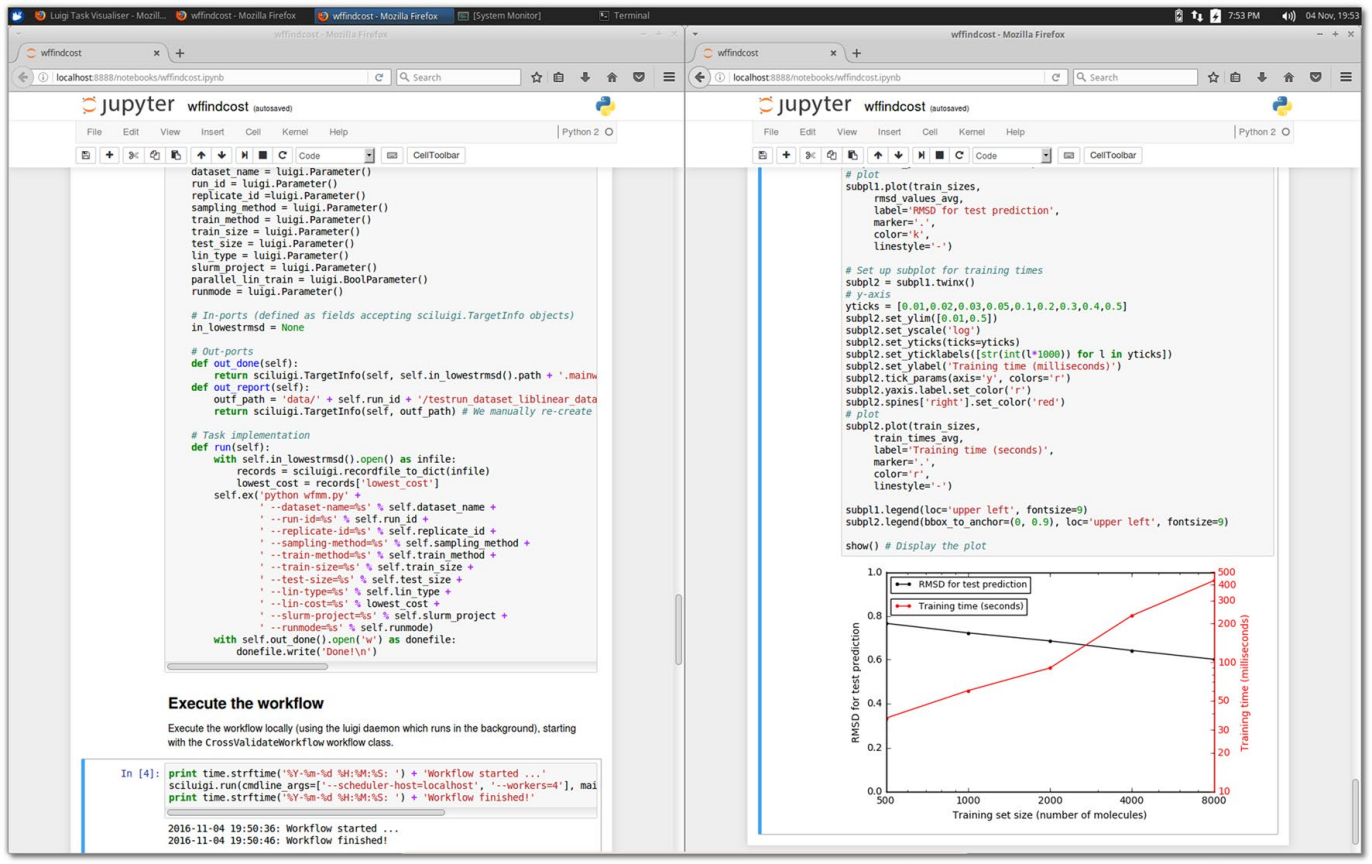
Jupyter notebook running the case study workflow. This *screenshot* shows the case study workflow running in a Jupyter notebook inside the virtual machine provided with the case study. The same Jupyter notebook is shown in two web browser windows arranged horizontally to enable showing multiple parts of the notebook in the same image. The *left side* of the image shows part of the workflow definition code, while the *right side* shows part of code for plotting the resulting values from the workflow run, and the resulting plot of RMSD values and training times, plotted against data set sizes

The plot in Fig. 6 shows the execution time and performance for the training set sizes tested. The best cost values vary between 0.05 and 0.1 for the different training set sizes.

#### Discussion and conclusion

From the plot in Fig. 6 it can be seen that this modelling approach does not work very well for really small data sets. This type of problem is greatly simplified using a workflow system with its workflow definition separated from task definitions and named ports, which allows connecting each input and output of each task independently.

**Fig. 7:**
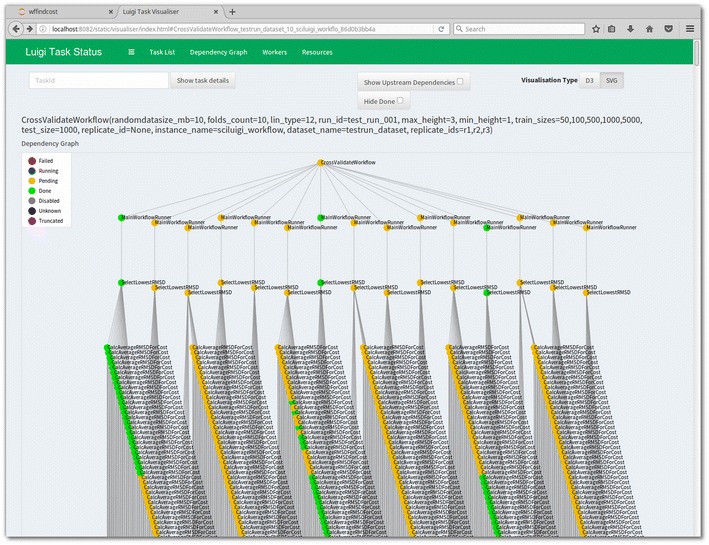
Dependency graph shown in Luigi’s web based visualiser. The dependency graph shows the structure of part of a workflow consisting of cross-validation fold generation combined with multiple parameter sweeps, which generates the large number of tasks, represented by *yellow*, *green* or *blue dots* in the image. Note that in this visualization, “downstream” tasks are at the *top*, while the “upstream” tasks are *below*. The number of tasks in this particular workflow is over 6000

This case study is a subset of a larger study carried out previously [34], which made use of 9 different training set sizes and also encompassed more computationally demanding modelling using LibSVM with the RBF kernel. As a runnable demonstration of the workflow, we also provide a virtual machine with a complete setup including a Jupyter [35, 36] notebook for running and replicating the case study. This virtual machine is available as a pre-made image at [37], while the code for creating the virtual image, including the workflow code and all dependencies, is available at [38]. See Fig. 6 for a screenshot of the Jupyter notebook where the case study workflow is being executed. Managing such large quantities of jobs (see Fig. 7) and resulting files in a fault-tolerant manner without a workflow management system is not a feasible approach. With the SciLuigi solution, the study could be carried out successfully on a shared computer cluster at the university high-performance computing centre [34]. The complete workflow of this previous study is available at GitHub [39].

## Discussion

The principle of *separate network definition*—the idea that connections are defined externally to the processes—is central to the flow-based programming paradigm, as it enables re-combining processes without changing their internals. This together with the idea of *named ports* for the inputs and outputs of processes, has proven very relevant in scientific workflow design as it allows an iterative, exploratory usage pattern which is common during the course of the scientific process. In the presented approach, these design principles have been applied in an improved API on top of the Luigi framework and demonstrated in workflow design for a machine learning problem in drug discovery. The presented approach, based on flow-based programming design principles, has turned out to make workflow code more manageable and less error-prone while also requiring drastically fewer code changes when adding new tasks to workflows.

In fact, workflows could now be defined that we were unable to create in vanilla Luigi, due to the prohibitively complex workarounds needed to get them to work in vanilla Luigi. HPC support and a detailed audit trail were also found to be important for the sample application. In summary, the modelling efforts referred to would simply not have been possible to perform with Luigi without the development and application of the SciLuigi solution.

Based on these experiences, we want to stress the importance in scientific workflows to (a) separate workflow connectivity information from task implementations and (b) allow connections between inputs and outputs of tasks to be defined separately. In other words, dependencies should be defined in terms of data rather than directly between tasks in order to capture all the information that constitute the workflow definition and which might change between workflow runs. If this is not done, one might end up in a situation where tasks have to be rewritten for each run, meaning that tasks are no longer fully re-usable. We also note that dependencies between tasks can always be inferred by the dependencies in the data produced by those tasks, so no information will be lost by defining dependencies in terms of the data.

By wrapping the separated workflow definition in its own task, we were able to solve the problem of duplicated parameters. Together with the separated network definition, this was found to result in a more agile and flexible way of designing and implementing complex machine learning workflows.

The fact that Luigi is a programming library has provided both benefits and drawbacks. A major advantage is that very complex workflow constructs can be constructed relatively easily, such as workflows that nest multiple parameter searches and cross-validation fold creation. For example, workflows with extensive, nested branchings, like in the aforementioned QSAR study [34], can be naturally defined in SciLuigi by creating nested for-loops that instantiate the tasks, with one for-loop per branch-point in the workflow, be it a parameter sweep, cross-validation construct or something else. See an example of this from a real-world problem in [40]. A drawback of Luigi being a programming library is that it does not come packed with all the convenience features that most WMS tools implemented as DSLs have, such as built-in audit logging and HPC resource manager integration. At the same time, being a programming library meant that it was relatively easy to work around the problems and limitations by extending it with the desired functionality without modifying the Luigi core library.

In the QSAR study [34], the audit feature of SciLuigi has turned out to be crucial for tracking errors and identifying mistakes in the workflow design as early as possible, and thus greatly helped to enable agile workflow design.

Finally we note that workflows including tasks with a dynamic number of outputs supposed to be routed to different downstream tasks, is still an area with room for improvement. Such workflows can be handled by SciLuigi as long as the number of outputs can be calculated or retrieved in the workflow definition code—that is, in the scheduling phase of the workflow execution. On the other hand, if the number of outputs and subsequent number of downstream tasks to be instantiated can not be calculated at the time of scheduling the workflow, SciLuigi can not model this in a natural way. This situation can show up for example when splitting a data set of unknown size into chunks of a defined size or when reading data from a database and creating one new task per result row, both of which are not uncommon scenarios for workflows in drug discovery. We thus identify this as an important area for further research.

## Conclusions

We present an approach for agile predictive modelling, combining design principles from flow-based programming with a workflow system. The developed SciLuigi library supports analysis of large data sets involving complex workflows with nested cross-validation and parameter sweeps, orchestrated on high-performance e-infrastructures. We envision that the approach will support data scientists in training and assessing machine learning models in drug discovery and related fields. The authors are aware of one company working in drug discovery testing out SciLuigi [41]. As of this writing, the library has been bookmarked—or “star-marked”—93 times on GitHub and forked 20 times [42], with at least 6 users sending in patches or suggestions for improvements.

## Methods

### QSAR modelling

In quantitative structure-activity relationships (QSAR) molecular properties are modelled by describing molecules numerically using molecular descriptors and correlating these values to the properties [43]. A common use case is to, by supervised machine learning, predict properties of molecules for which these properties are unknown. First the molecular descriptors are calculated and then a predictive model is constructed based on a set of known data. The model is said to “learn” or to “be trained”. The set of known data is often called a *training set* and the bigger the training set the better are the chances of getting a good model, i.e., the model has seen enough examples to adequately cover the relevant chemical space.

For large data sets, the calculation of the molecular descriptors can require quite a lot of CPU time and so can the construction of the predictive model. Execution time of the descriptor calculation tends to increase linearly with increased training set size but the relationship between execution time for model building and training set size depends on the modelling approach. However, in general, better models require larger training sets, which in turn require more CPU hours. Many different molecular descriptors are in common use. We used molecular signatures [32] which have proved to work well [44–46].

Often, the QSAR model algorithms come with free parameters that need to be determined, e.g., support vector machines based on the radial basis function has the free parameters $\gamma$ and *cost* [47] and k-nearest neighbour has *k* [46]. A common way of determining actual values for parameters such as these is a grid search or “parameter sweep”. A predetermined range of candidate values are tested one by one on a part of the training set and evaluated using another part of the training set as reference. The value that makes the model perform the best is then used to build the final model. This is commonly done using *n*-fold cross-validation in which the training set is split into *n* parts (often 10) and each of these parts is used as reference once, while the remaining parts in each such iteration are merged together as the training set. In the case of 10-fold cross-validation this means that 10 estimates are created where each is based on 90% of the training data. Finally, the mean or median of the predicted values for the parameter can be used when building the final model.

### Luigi

Luigi is a batch workflow system written in Python and developed by the streaming music company Spotify, to help manage workloads of periodic analysis tasks like lists of top songs and artists for different periods of time. It is released as open source and freely available on GitHub [26].

Luigi is implemented as a programming library. In short, creating a task with its default API involves sub-classing the luigi.Task base class, adding fields for parameters and overriding a few class methods. Namely, the requires() method, which returns upstream tasks of the current task; the output() method, which returns all the outputs of the current task; and the run() method, which defines what the task does. An example definition of a task that depends on another task is available in Fig. 4.

Luigi provides automatic command-line interface generation based on parameters added to task classes. It also provides a central scheduler, a web-based workflow progress visualisation, logging facilities and a combination of support for Apache Hadoop [28], Apache Spark [29] and normal file systems in the same tool. Additionally, it includes a light-weight solution for distributing workloads across nodes without the need of a resource manager such as SLURM [48].

Luigi has certain characteristics which are problematic for complex use cases. A primary focus of Luigi is in situations with a relatively fixed workflow connectivity but with frequent variations in the parameter values, such as date ranges [49]. This could be contrasted with scientific exploration where the workflow connectivity often varies extensively as well. More specifically, the relevant problems in Luigi are:

1. Dependencies are defined inside a tasks’ definition. The problem with this approach is that tasks are not fully independent and re-usable since they need to be rewritten every time the workflow connectivity changes. This means that tasks can not be kept in a common task library and plugged into workflows when needed.
2. Dependencies are specified directly between tasks rather than between the inputs and outputs of tasks. This means that tasks need to know the names of outputs of upstream tasks and need to implement code for looking up the correct output. This again ties two tasks together by their very definition such that they are not fully self-contained and interchangeable with other tasks that consume and produce data of the same format. This can be exemplified by the implementation of a Luigi task in Fig. 4, lines 19 and 23, where we see that in the run() method of the downstream task, there is navigation code tied to the structure of an upstream task.
3. Parameter values in Luigi need to be provided each time a task is instantiated. This combined with the fact that tasks are instantiated inside downstream tasks during the scheduling phase, means that parameter values needed for a task’s instantiation also need to be known in all downstream tasks. In other words, the parameter value will need to be passed on all the way from the most downstream task of the workflow up to where it is actually used. This is illustrated in Fig. 3a.

The above shortcomings imply that Luigi tasks are not fully independent – in many cases they need to contain parameter definitions not used by themselves but only serving to be passed to upstream tasks. Thus, swapping out one task in a workflow will require rewriting not only this task but also all downstream tasks (illustrated in Fig. 4). This goes against the vision of agile ”pluggable”, component-oriented, iterative workflow construction, central to scientific computational use cases. However, this was not the primary focus when developing Luigi at Spotify [49].

### Flow-based programming

Flow-based programming (FBP) is a programming paradigm invented by John Paul Morrison at IBM in the late 1960’s to ease development of complex data processing programs in mainframe computers [50, 51]. It is a definition for applications in general but many of the design patterns apply equally well to workflows, which can be seen as a form of application. In short, FBP is a specialised form of the dataflow programming paradigm [52]. From dataflow, it takes the concept of “black box”, asynchronous processes which communicate via message passing over pre-defined connections. FBP adds the ideas of separate network definition, named in- and out-ports, channels with bounded buffers and information packets with defined lifetimes for the data exchange. From these principles, separate network definition and named ports are used in this study.

## Abbreviations

DSL: domain-specific language
FBP: flow-based programming
GUI: graphical user interface
HPC: high-performance computing
QSAR: quantitative structure–activity relationship
WMS: workflow management system
